# A Study of the Cross-Scale Causation and Information Flow in a Stormy Model Mid-Latitude Atmosphere

**DOI:** 10.3390/e21020149

**Published:** 2019-02-05

**Authors:** X. San Liang

**Affiliations:** Center for Ocean-Atmosphere Dynamical Studies, Nanjing Institute of Meteorology, 219 Ningliu Blvd, Nanjing 210044, China; x.san.liang@gmail.com

**Keywords:** causality, information flow, multiscale interaction, self-organization, storm, atmospheric jet stream, weather and climate patterns

## Abstract

A fundamental problem regarding the storm–jet stream interaction in the extratropical atmosphere is how energy and information are exchanged between scales. While energy transfer has been extensively investigated, the latter has been mostly overlooked, mainly due to a lack of appropriate theory and methodology. Using a recently established rigorous formalism of information flow, this study attempts to examine the problem in the setting of a three-dimensional quasi-geostrophic zonal jet, with storms excited by a set of optimal perturbation modes. We choose for this study a period when the self-sustained oscillation is in quasi-equilibrium, and when the energetics mimick the mid-latitude atmospheric circulation where available potential energy is cascaded downward to smaller scales, and kinetic energy is inversely transferred upward toward larger scales. By inverting a three-dimensional elliptic differential operator, the model is first converted into a low-dimensional dynamical system, where the components correspond to different time scales. The information exchange between the scales is then computed through ensemble prediction. For this particular problem, the resulting cross-scale information flow is mostly from smaller scales to larger scales. That is to say, during this period, this model extratropical atmosphere is dominated by a bottom-up causation, as collective patterns emerge out of independent entities and macroscopic thermodynamic properties evolve from random molecular motions. This study makes a first step toward an important field in understanding the eddy–mean flow interaction in weather and climate phenomena such as atmospheric blocking, storm track, North Atlantic Oscillation, to name a few.

## 1. Introduction

The atmospheric motion is rich in scale. In many cases, the formation of weather and climate patterns can be attributed to the interaction between a few scale ranges such as that between synoptic eddies and the jet stream, or that among the synoptic eddies, the low-frequency variability, and the mean flow, just as the storm track in the Northern Hemisphere (e.g., [1,2,3]), the blocking high (e.g., [4,5]), the sudden stratospheric warming, e.g., [6,7], and references therein), the North Atlantic Oscillation (e.g., [8,9,10,11,12,13]), to name but a few. This makes multiscale interaction a central issue in dynamic meteorology.

An important problem in multiscale interaction is how energy is transferred across scales; this transfer is closely related to the fundamental processes in atmospheric flows, namely, barotropic instability and baroclinic instability. The extratropical atmosphere is special in that, while on the whole the available potential energy is cascaded toward smaller scales, the kinetic energy is inversely transferred upward to larger scales (e.g., [14,15]). More specifically, there exists a symbiotic relationship between the synoptic-scale and planetary-scale disturbances. In a 3-scale setting, Cai and Mak [1] found that the former are produced and maintained through extracting energy from the zonal flow via Reynolds stress; they then supply energy to the latter through upscale energy transfer, while the latter form regions of enhanced baroclinicity where the former preferentially grow. This kind of energetic cycle has been employed to interpret many weather/climate phenomena. In the case of atmospheric blockings, for example, Holopainen and Fortelius [16] identified an enhanced transfer of eddy kinetic energy (KE) to the mean flow over the storm tracks. Hansen and Chen [17] found that the nonlinear interaction between the cyclone-scale and planetary-scale waves is essential to the Atlantic blocking, while baroclinic amplification plays the most important role in the formation of the Pacific one. After the maintenance stage, the eddy kinetic energy is converted back to the eddy available potential energy (APE), leading to the decay of the event [18].

Another example is the inverse relationship between the boreal wintertime Pacific jet strength and storm-track intensity. It has long been observed that, in winters when the jet stream over the North Pacific is extremely strong, the storm track is however unexpectedly weak. This observation, which is at odds with the prediction of the classical linear baroclinic instability theory, has caught enormous attention from the atmospheric community. For example, based on energy budget diagnostics, Chang [19] and Nakamura and Sampe [20] found that, in that case, the synoptic waves tend to be trapped in the upper troposphere and hence are less efficient to tap APE from the background baroclinicity. In some other studies, it has been argued (e.g., [21,22]) that the inverse KE transfer may suppress the APE release, and hence contribute to the inverse relationship. Considering that atmospheric processes, cyclogenesis in particular, tend to be localized in space and time, Liang and Robinson [23] established, on the basis of the multiscale window transform of Liang and Anderson [24], a methodology for local multiscale atmospheric energetics analysis. The resulting interscale energy transfers turn out to bear a Lie bracket form, reminiscent of the Poisson bracket in Hamiltonian dynamics, and, furthermore, it naturally has the property that the energy redistributed among scales remains conserved (see [25]). To distinguish them, these energy transfers have been called “canonical transfers” ever since. With them, it is shown that, at each spatial location, there exists a local Lorenz cycle which allows one to trace the origin of the observed, if any, energy burst processes. Recently, by analyzing the local Lorenz cycles, it is found that, in boreal winters, the storms over the North Pacific actually are generated at latitudes far northward of the jet core [26]. This greatly lowers the relevance of the jet strength to the storm-track intensity, and hence the inverse relationship is actually not much a surprise. That is to say, the linear baroclinic instability theory may still hold [27], and the inverse relationship should be attributed to internal dynamics.

Energy and entropy are two parallel basic notions in physics. Naturally, the transfer of entropy, i.e., information transfer or information flow, across scales must make another fundamental problem in multiscale interaction. This problem, however, has been mostly overlooked in the past, mainly due to a lack of appropriate theory and research methodology. (So far, only a few studies have touched this issue, e.g., [28,29]). In physics, entropy may make an objective functional to be optimized to determine or regulate the distribution of energy. Without knowledge of the information flow across scales, the understanding of multiscale interaction is then incomplete. For example, in a classical energetics analysis, the emergence of a structure on another scale is driven by Reynolds stress or Reynolds stress-like quantities, which are essentially correlations between perturbation fields (e.g., [30]). While causation implies correlation, correlation does not necessarily imply causation ([31]; see below in Section 2. Thus, it is likely that an interaction which has a two-way energy flow may in fact have a one-way causation (as the example in the following; see below). We hence want to give this an investigation, in the hope of shedding some light on the dark side of the multiscale interaction problem. We emphasize that this is just the first step to exploring a rather profound field; it is by no means our intention to solve all the problems. In the following we first give a brief introduction of a recently developed theory of information flow, then introduce the atmospheric model (Section 3) and its solution. The model is reduced approximately to a low-dimensional dynamical system (Section 4), and with it the information flows across the scales are computed through ensemble prediction (Section 5). As we will see, remarkably, the system nearly has a bottom-up causation, consistent with how a reductionist in evolutionary biology would view the emergence of a higher-level organization out of independent, lower-level entities. This study is summarized in Section 6.

## 2. A Brief Review of the Theory of Causation and Information Flow That Pertains to This Study

Causal inference is a problem lying at the heart of science. In many disciplines, it makes a direct research objective. It is also an important topic in philosophy, as it forms a “guide to higher understanding” [32]. However, it is very challenging; in fact, it has been identified as “one of the biggest challenges” in the science of big data [33]. During the past thirty years, it has come to a consensus that the widely applicable notion in physics, namely, information flow or information transfer as it may appear in the literature, is logically associated with causality: The latter is the key to the former, while the former provides not only the magnitude but also the direction for the latter. Realizing that information flow is a real physical notion, Liang and Kleeman [34] put the problem on a rigorous footing, and obtained in a closed form the information flow between the components of a 2D dynamical system. This formalism was soon generalized by Majda and Harlim [35] to a setting with two subspaces. Recently, it was successfully extended by Liang [36] to systems of arbitrary dimensionality. The following is a brief review of the work that pertains to this study.

We begin by stating a principle or an observational fact about causality:

If the evolution of an event, say, $X_{1}$, is independent of another one, $X_{2}$, then the causality from $X_{2}$ to $X_{1}$ is zero.

Since it is the only quantitatively stated fact about causality, all previous empirical/half-empirical causality formalisms have attempted to verify it in applications. Considering its importance, it has been referred to as the **principle of nil causality** [36]. Recently, Smirnov [37] systematically examined the traditional formalisms, i.e., transfer entropy analysis and/or Granger causality testing, and found that they cannot verify the principle in a wide range of situations; similar conclusions have been drawn by Lizier and Prokopenko [38]. We will see soon below that, within our framework, this principle turns out to be a proven theorem.

Now, consider an *n*-dimensional continuous-time stochastic system for state variables $x=(x_{1},\ldots ,x_{n})$

(1) $$\begin{array}{c} \frac{dx}{dt}=F\left(x,t\right)+B\left(x,t\right)\dot{w}, \end{array}$$

where $F=(F_{1},\ldots ,F_{n})$ may be arbitrary nonlinear functions of x and *t*, $\dot{w}$ is a vector of white noise, and $B=(b_{ij})$ is the matrix of perturbation amplitudes which may also be any functions of x and *t*. Here, we adopt the convention in physics and do not distinguish deterministic and random variables; in probability theory, they are usually distinguished with capital and lower-case symbols. Assume that F and B are both differentiable with respect to x and *t*. We then have the following theorem [36]:

**Theorem** **1.** *For the system (1), the rate of information flowing from $x_{j}$ to $x_{i}$ (in nats per unit time) is*

(2) $$\begin{array}{ccc} T_{j\to i} & = & -E\left[\frac{1}{\rho_{i}}\int_{R^{n-2}}\frac{\partial (F_{i}\rho_{\setminus \;j})}{\partial x_{i}}dx_{\setminus \;i\setminus \;j}\right]+\frac{1}{2}E\left[\frac{1}{\rho_{i}}\int_{R^{n-2}}\frac{\partial^{2}\left(g_{ii}\rho_{\setminus \;j}\right)}{\partial x_{i}^{2}}dx_{\setminus \;i\setminus \;j}\right]\\ & = & -\int_{R^{n}}\rho_{j|i}\left(x_{j}|x_{i}\right)\frac{\partial (F_{i}\rho_{\setminus \;j})}{\partial x_{i}}dx+\frac{1}{2}\int_{R^{n}}\rho_{j|i}\left(x_{j}|x_{i}\right)\frac{\partial^{2}\left(g_{ii}\rho_{\setminus \;j}\right)}{\partial x_{i}^{2}}dx, \end{array}$$

*where $dx_{\setminus \;i\setminus \;j}$ signifies $dx_{1}\ldots dx_{i-1}dx_{i+1}\ldots dx_{j-1}dx_{j+1}\ldots dx_{n}$, E stands for mathematical expectation, $g_{ii}=\sum_{k=1}^{n}b_{ik}b_{ik}$, $\rho_{i}=\rho_{i}\left(x_{i}\right)$ is the marginal probability density function (pdf) of $x_{i}$, $\rho_{j|i}$ is the pdf of $x_{j}$ conditioned on $x_{i}$, and $\rho_{\setminus \;j}=\int_{R}\rho \left(x\right)dx_{j}$.*

If $T_{j\to i}=0$, then $x_{j}$ is not causal to $x_{i}$; otherwise, it is causal, and the absolute value measures the magnitude of the causality from $x_{j}$ to $x_{i}$. For discrete-time mappings, the information flow is in a much more complicated form; see [36].

**Corollary** **1.**  [39] *When n=2,*

(3) $$\begin{array}{c} T_{2\to 1}=-E\left[\frac{1}{\rho_{1}}\frac{\partial (F_{1}\rho_{1})}{\partial x_{1}}\right]+\frac{1}{2}E\left[\frac{1}{\rho_{1}}\frac{\partial^{2}(g_{11}\rho_{1}}{\partial x_{1}^{2}})\right]. \end{array}$$

In the absence of noise, this is precisely the result of [34] based on a heuristic argument.

There is a nice property for the above information flow:

**Theorem** **2.** (Principle of nil causality) If in Equation (1) neither $F_{1}$ nor $g_{11}$ depends on $X_{2}$, then $T_{2\to 1}=0$.

Note that this is precisely the principle of nil causality. Remarkably, here it appears as a proven theorem, while the classical ansatz-like formalisms fail to verify it in many problems (e.g., [37]).

In the case with only two time series (no dynamical system is given), we have the following result [31]:

**Theorem** **3.** *Given two time series $X_{1}$ and $X_{2}$, under the assumption of a linear model with additive noise, the maximum likelihood estimator (mle) of the rate of information flowing from $X_{2}$ to $X_{1}$ is*

(4) $$\begin{array}{c} {\hat{T}}_{2\to 1}=\frac{C_{11}C_{12}C_{2,d1}-C_{12}^{2}C_{1,d1}}{C_{11}^{2}C_{22}-C_{11}C_{12}^{2}}, \end{array}$$

*where $C_{ij}$ is the sample covariance between $X_{i}$ and $X_{j}$, and $C_{i,dj}$ the sample covariance between $X_{i}$ and a series derived from $X_{j}$ using the Euler forward differencing scheme: ${\dot{X}}_{j,n}=\left(X_{j,n+k}-X_{j,n}\right)/\left(k\Delta t\right)$, with k≥1 some integer.*

Equation (4) is rather concise in form; it only involves the common statistics, i.e., sample covariances. In other words, a combination of some sample covariances will give a quantitative measure of the causality between the time series. This makes causality analysis, which otherwise would be complicated with the classical empirical/half-empirical methods, very easy. Nonetheless, note that Equation (4) cannot replace (3); it is just the mle of the latter. A statistical significance test must be performed before a causal inference is made based on the computed $T_{2\to 1}$. For details, refer to [31].

Considering the long-standing debate ever since George Berkeley in 1710 over correlation versus causation, we may rewrite (4) in terms of linear correlation coefficients, which immediately implies [31]:

Causation implies correlation, but correlation does not imply causation.

In fact, suppose there is no correlation between $X_{1}$ and $X_{2}$, $C_{12}=0$; then, by Equation (4), ${\hat{T}}_{2\to 1}=0$. However, from ${\hat{T}}_{2\to 1}=0$, one cannot come up with $C_{12}=0$.

Causality can be normalized in order to reveal the relative importance of a causal relation. However, the normalization is by no means as trivial as that for covariance, considering that information flow is asymmetric in direction ($T_{2\to 1}\neq T_{1\to 2}$ in general), and, in addition, there is no such property like a Cauchy–Schartz inequality that makes it possible for covariance to be normalized. In [40], a way of normalization is given, but a complete solution is yet to be sought.

The above formalism has been validated with many benchmark systems (e.g., [36]) such as Baker transformation, Hénon map, Kaplan–Yorke map, and Rössler system, to name a few. Particularly, Equation (4) has been validated with touchstone problems where the traditional Granger causality test and transfer entropy analysis fail. An example is the highly chaotic anticipatory system problem described in [41], which with Equation (4) turns out not to be a problem at all.

The formalism has been successfully applied to the studies of many real world problems, among them are the causal relation between El Niño-Indian Ocean Dipole [31], tropical cyclone genesis prediction [42], near-wall turbulence [28], global climate change ([43,44]), and financial time series analysis [40], to name but a few. Here, we particularly want to mention the study by Stips et al. [43] who, through examining with Equation (4) the causality between the CO${}_{2}$ index and the surface air temperature, identified a reversing causal relation with time scale. They found, during the past century, that CO${}_{2}$ emission indeed drives the recent global warming; the causal relation is one-way, i.e., from CO${}_{2}$ to global mean atmosphere temperature. Moreover, they were able to find how the causality is distributed over the globe, thanks to the quantitative nature of our formalism. However, on a time scale of 1000 years or over, the causality is completely reversed; that is to say, on a paleoclimate scale, it is global warming that causes CO${}_{2}$ concentration to rise!

## 3. A Quasi-Geostrophic Atmospheric Model

### 3.1. The Governing Equation

Consider a three-dimensional (3D) quasi-geostrophic (QG) model on a β-plane within a channel between latitudes y=±1 (cf. [45,46]):

(5) $$\begin{array}{c} \frac{\partial \zeta }{\partial t}+J\left(\psi ,\zeta \right)+\beta \frac{\partial \psi }{\partial x}=-r\zeta +A_{H}{\nabla_{H}}^{2}\zeta , \end{array}$$

where ψ is streamfunction,

(6) $$\begin{array}{c} \zeta ={\nabla_{H}}^{2}\psi +\frac{\partial \;}{\partial z}\left(\frac{F_{r}^{2}}{N^{2}}\frac{\partial \psi }{\partial z}\right) \end{array}$$

potential vorticity, *J* the Jacobian operator such that $J\left(\psi ,\zeta \right)=\frac{\partial \psi }{\partial x}\frac{\partial \zeta }{\partial y}-\frac{\partial \zeta }{\partial x}\frac{\partial \psi }{\partial y}$, *N* the buoyancy frequency, and $F_{r}$ the rotational internal Froude number. On the right-hand side, the first term stands for the Rayleigh-type friction and the second is the horizontal dissipation of the potential vorticity. In this study, these two terms are set to be zero ($r=A_{H}=0$). This equation together with the following boundary conditions:

(7) $$\begin{array}{c} \psi =\mathrm{const}\;y=\pm 1, \end{array}$$

(8) $$\begin{array}{c} \psi \;\mathrm{is}\;\mathrm{periodic}\;\mathrm{in}\;x, \end{array}$$

(9) $$\begin{array}{c} \frac{\partial \;}{\partial t}\left(\frac{F_{r}^{2}}{N^{2}}\frac{\partial \psi }{\partial z}\right)+J(\psi ,\left(\frac{F_{r}^{2}}{N^{2}}\frac{\partial \psi }{\partial z}\right)=\left\{\begin{array}{cc} 0,\; & z=1,\\ -J(\psi ,b), & z=0, \end{array}\right. \end{array}$$

where *b* is the bottom relief, forms the problem that we are about to solve. In Equation (9), at z=1, the atmosphere is taken as a rigid lid (vertical velocity w(z=1)=0); at z=0, a flat bottom is considered and hence b=0.

We choose to solve the equation for the perturbation around the mean state $\bar{\psi }$. The mean state is a zonally homogeneous jet

(10) $$\begin{array}{c} \bar{U}=\bar{U}\left(y,z\right). \end{array}$$

It is easy to verify that it satisfies the QG equation for an ideal fluid. Here, $\bar{U}$ horizontally is assumed to be a cosine jet within [−L,L], L≤1, as that in [47],

(11) $$\begin{array}{c} \bar{U}=Z\left(z\right)\cdot \cos^{2}\frac{\pi y}{2L}=\frac{1+\cos\frac{\pi }{L}y}{2}\cdot Z\left(z\right),\;y\in \left[-L,L\right]; \end{array}$$

outside [−L,L], the fluid is motionless. In this study, *L* is chosen to be 1, Z(z) is prescribed such that $\frac{\partial \bar{U}}{\partial z}=0$ near z=0 and z=1, and is maximized in the upper troposphere. From $\bar{U}$, the mean states

(12) $$\begin{array}{c} \bar{\psi }\left(y,z\right)=-\int_{-1}^{y}\bar{U}\left(y,z\right)dy, \end{array}$$

(13) $$\begin{array}{c} \bar{\zeta }\left(y,z\right)={\bar{\psi }}_{yy}+\frac{\partial \;}{\partial z}\left(\frac{F_{r}^{2}}{N^{2}}\frac{\partial \bar{\psi }}{\partial z}\right) \end{array}$$

can be easily obtained, and

(14) $$\begin{array}{c} \frac{\partial \bar{\zeta }}{\partial y}=-\frac{\partial S}{\partial z}\frac{\partial \bar{U}}{\partial z}-S\frac{\partial^{2}\bar{U}}{\partial z^{2}}-\frac{\partial^{2}\bar{U}}{\partial y^{2}}=-S_{z}{\bar{U}}_{z}-S{\bar{U}}_{zz}-{\bar{U}}_{yy}, \end{array}$$

where we have shortened $F_{r}^{2}/N^{2}$ as S(z).

Let $\psi =\bar{\psi }+\psi^{\prime }$, $u=\bar{U}+u^{\prime }$, $\zeta =\bar{\zeta }+\zeta^{\prime }$. The perturbation equation is

(15) $$\begin{array}{c} \frac{\partial \zeta^{\prime }}{\partial t}+\left(\bar{U}+u^{\prime }\right)\frac{\partial \zeta^{\prime }}{\partial x}+v^{\prime }\frac{\partial \zeta^{\prime }}{\partial y}+\left[\beta -\left(S_{z}{\bar{U}}_{z}+S{\bar{U}}_{zz}-{\bar{U}}_{yy}\right)\right]\frac{\partial \psi^{\prime }}{\partial x}=0. \end{array}$$

Correspondingly, the boundary conditions are changed to: $\psi^{\prime }$ vanishes at y=±1, and

(16) $$\begin{array}{c} \frac{\partial S\psi_{z}^{\prime }}{\partial t}+\left(\bar{U}+u^{\prime }\right)\frac{\partial (}{\partial z}S\psi_{z}^{\prime })+v^{\prime }S{\bar{U}}_{z}+v^{\prime }\frac{\partial S\psi_{z}^{\prime }}{\partial y}=0 \end{array}$$

at z=0,1. Note in this study ${\bar{U}}_{z}=0$ at z=0,1, hence the third term vanishes. Thus, this is simply the horizontal advection of $S\psi_{z}^{\prime }$. If initially $\psi_{z}^{\prime }=0$, and there is no energy flux toward the upper and lower boundaries, it will remain unperturbed. To simplify the problem, we then set the vertical boundary condition as

(17) $$\begin{array}{c} \frac{\partial \psi^{\prime }}{\partial z}=0,\;\mathrm{at}\;z=0,1. \end{array}$$

As we will see later, though with such a rather strong condition, the result does reproduce the expected energetics typical of the large-scale mid-latitude atmospheric motion.

### 3.2. Model Setup

In this study, we choose a mesh with spacings of Δx=0.2, Δy=0.04, and Δz=0.2, which results in a grid with 50×51×5 points. Choose a vertical profile for the basic flow $Z\left(z_{k}\right)=\left(0.2,0.2,0.6,1,1\right)$ such that $\partial \bar{U}/\partial z=0$ at z=0 (k=1) and z=1 (k=5). To determine $S\left(z\right)=F_{r}^{2}/N^{2}$, first notice that $F_{r}=f_{0}L_{0}/N_{0}H_{0}$ is the rotational Froude number; usually, it is taken as 1. We hence only need to pay attention to the buoyancy frequency *N*. Scale it by $N_{0}$. In dimensional form, it is defined as

(18) $$\begin{array}{c} N_{0}N={\left(-\frac{g}{\bar{\rho }}\frac{\partial \bar{\rho }}{\partial z}\right)}^{1/2}, \end{array}$$

where ρ is the density of the fluid. While for oceans this can be directly computed, for the atmosphere, it is usually converted into another form

(19) $$\begin{array}{c} N_{0}N={\left(\frac{g}{\bar{\theta }}\frac{\partial \bar{\theta }}{\partial z}\right)}^{1/2}={\left(g\frac{\partial \log\bar{\theta }}{\partial z}\right)}^{1/2}. \end{array}$$

Here, $\bar{\theta }$ is potential temperature; it is the temperature of an air parcel moving adiabatically to some reference pressure (usually 1000 hPa), i.e., a temperature with the effect of pressure change excluded:

(20) $$\begin{array}{c} \bar{\theta }=\bar{T}{\left(\frac{P_{0}}{\bar{P}}\right)}^{R/c_{p}}\equiv \bar{T}{\left(\frac{1000}{\bar{P}}\right)}^{\kappa_{d}}, \end{array}$$

where $\kappa_{d}\approx 0.286$, R=8.314J/mol·K=287J/kg·K is the ideal gas constant, and $c_{p}=1004\;J/\mathrm{kg}\cdot K$ the specific heat capacity at constant pressure. This yields

(21) $$\begin{array}{c} N_{0}^{2}N^{2}=\frac{g}{\bar{T}}\left(\frac{\partial \bar{T}}{\partial z}+\frac{g}{c_{p}}\right), \end{array}$$

where $-\frac{\partial \bar{T}}{\partial z}$ is the lapse rate.

For the troposphere, usually the lapse rate can be roughly taken as a constant $\frac{\partial \bar{T}}{\partial z}\approx -0.65\;{}^{\circ }C/100\;m=6.5\times 10^{-3}\;{}^{\circ }C/m$. That is to say,

(22) $$\begin{array}{c} \bar{T}={\bar{T}}_{0}-6.5\times 10^{-3}z. \end{array}$$

Thus,

(23) $$\begin{array}{ccc} N^{2}\left(z\right) & = & \frac{g}{\bar{T}}\left(\frac{\partial \bar{T}}{\partial z}+\frac{g}{c_{p}}\right)\\ & \approx & \frac{0.032}{{\bar{T}}_{0}-6.5\times 10^{-3}z}. \end{array}$$

**Figure 1:**
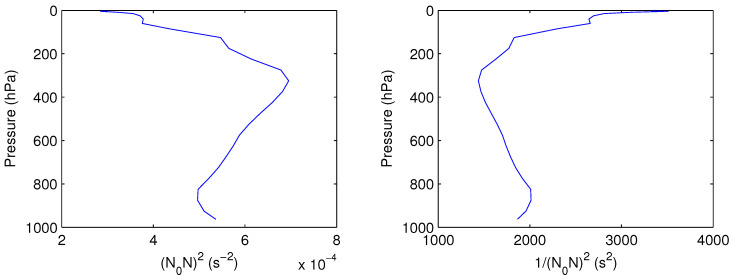
Vertical profile of $N_{0}^{2}N^{2}$ for the atmosphere between $20{}^{\circ }\;N$–$60{}^{\circ }\;N$.

In Figure 1, the vertical profile of $N_{0}^{2}N^{2}$ for the atmosphere between latitudes $20{}^{\circ }\;N$–$60{}^{\circ }\;N$ is computed. Furthermore, its reciprocal $N_{0}^{-2}N^{-2}$ is shown.

By Figure 1, $N_{0}^{2}N^{2}$ varies from $5\times 10^{-4}$$s^{-2}$ near the bottom to $7\times 10^{-4}$$s^{-2}$ at tropopause. If normalized by $N_{0}^{2}$, this means it varies from 1 to 1.4. Considering that the lapse rate $-\frac{\partial \bar{T}}{\partial z}$ is nearly a constant 0.65 ${}^{\circ }C/100\;m$, $N_{0}^{2}/N^{2}=S\left(z\right)$ ($F_{r}^{2}=1$) and hence almost decreases linearly with *z*, with a rate of $(1-\frac{1}{1.4})/5=0.057$ per level. In a brief summary, Table 1 gives a list of the parameters for the model.

### 3.3. Solution Strategy

The problems (15)–(17) is solved with a leap-frog scheme. To suppress the computational mode that may arise due to the time splitting, the result at each integration step is filtered in time as follows:

(24) $$\begin{array}{c} \psi^{n}=\left(1-\alpha \right)\psi^{n}+\frac{\alpha }{2}\left(\psi^{n+1}+\psi^{n-1}\right) \end{array}$$

with a weak filter coefficient α=0.01. This is equivalent to a weak dissipation of the flow. At each step, it is required to solve the 3D elliptic equation

(25) $$\begin{array}{c} {\nabla_{H}}^{2}\psi +\frac{\partial \;}{\partial z}\left(S\left(z\right)\frac{\partial \psi }{\partial z}\right)=\zeta \end{array}$$

for ψ subject to

(26) $$\begin{array}{c} \left\{\begin{array}{c} \psi_{z}\left(z=1\right)=0,\\ \psi_{z}\left(z=0\right)=0, \end{array}\right. \end{array}$$

in the vertical, a no-flux condition in *y*, and a periodic condition in *x*. We may separate *z* from (x,y) to convert the 3D equation to a set of 2D equations. Separation of variables results in an eigenvalue problem

(27) $$\begin{array}{c} \frac{d\;}{dz}\left(S\left(z\right)\frac{d\theta_{k}\left(z\right)}{dz}\right)=\lambda_{k}\theta_{k}\left(z\right) \end{array}$$

together with a boundary condition

(28) $$\begin{array}{c} \frac{d\theta_{k}}{dz}=0,\;\mathrm{at}\;\;z=0,\;1. \end{array}$$

This is a Sturm–Liouville problem (cf. [48]), and it can be proven that the resulting eigenvectors $\left\{\theta_{k}\right\}$ form an orthogonal set. The set is complete and can be normalized. Thus, it can be made as an orthonormal basis. Expand $\psi^{\prime }$ and $\zeta^{\prime }$ (time-dependence suppressed for notational simplicity) with the basis:

(29) $$\begin{array}{c} \psi^{\prime }\left(x,y,z\right)=\sum_{k=1}^{n}{\tilde{\Phi }}_{k}\left(x,y\right)\cdot \theta_{k}\left(z\right), \end{array}$$

(30) $$\begin{array}{c} \zeta^{\prime }\left(x,y,z\right)=\sum_{k=1}^{n}{\tilde{Z}}_{k}\left(x,y\right)\cdot \theta_{k}\left(z\right), \end{array}$$

where *n* is the number of levels (of the discretized model) in the vertical direction, and substitute them into the original Equation (25) to get

(31) $$\begin{array}{c} \sum_{k=1}^{n}{\nabla_{H}}^{2}{\tilde{\Phi }}_{k}\cdot \theta_{k}\left(z\right)+\sum_{k=1}^{n}{\tilde{\Phi }}_{k}\frac{\partial \;}{\partial z}\left[S\left(z\right)\frac{\partial \theta_{k}\left(z\right)}{\partial z}\right]=\sum_{k=1}^{n}{\tilde{Z}}_{k}\left(x,y\right)\theta_{k}\left(z\right). \end{array}$$

By the orthonormality of $\left\{\theta_{k}\right\}$, the original 3D equation is transformed into *n* decoupled 2D equations:

(32) $$\begin{array}{c} {\nabla_{H}}^{2}{\tilde{\Phi }}_{k}+\lambda_{k}{\tilde{\Phi }}_{k}={\tilde{Z}}_{k}, \end{array}$$

which can be solved individually.

**Figure 2:**
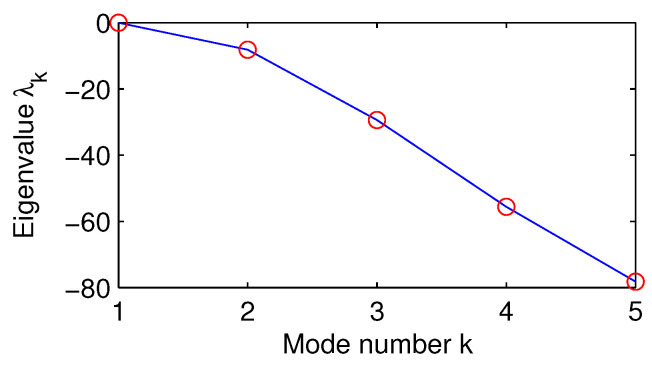
The computed eigenvalues $\lambda_{k}$.

**Figure 3:**
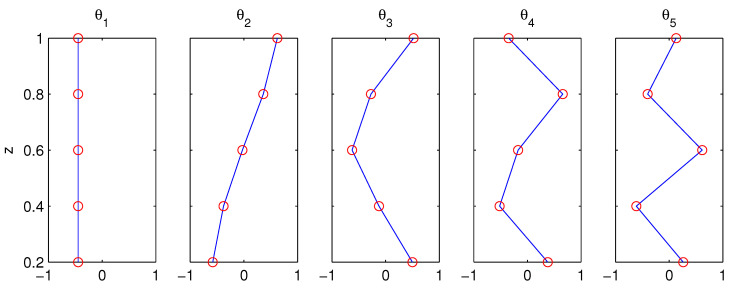
The eigenvectors $\theta_{k}\left(z\right)$ corresponding to the eigenvalues $\lambda_{k}$ in Figure 2.

The eigenvalue problems (27)–(28) are solved with the parameters as listed in Table 1. The resulting eigenvalues $\lambda_{k}$, k=1,…,5, are all negative; see Figure 2. The corresponding eigenvectors $\theta_{k}$ are displayed in Figure 3; it is easy to verify that they are orthonormal (cf. [48]).

When $N^{2}$ is constant, the problem can be solved analytically (e.g., [49]). In that case, the most rudimentary mode is barotropic and the remaining ones baroclinic. In this case, $\theta_{1}$ is the very barotropic mode, and $\theta_{3}$, $\theta_{4}$, $\theta_{5}$ are approximately sinusoidal and hence are just like the baroclinic modes. Here, as $N^{2}$ varies with height, mode 2 somehow has a different form. Note that $c_{k}=1/\sqrt{-\lambda_{k}}$ corresponds to the phase speed of mode k.

### 3.4. Initialization

Different initial disturbances will in general yield different solutions, many of which may be eventually damped out. In order to obtain a quasi-equilibrium oscillatory state, there exists some “optimal perturbation.” To find this, linearize Equation (15) to get

(33) $$\begin{array}{ccc} \frac{\partial \zeta^{\prime }}{\partial t} & = & -\bar{U}\frac{\partial \;}{\partial x}\left[{\nabla_{H}}^{2}\psi^{\prime }+\frac{\partial \;}{\partial z}\left(S\frac{\partial \psi^{\prime }}{\partial z}\right)\right]-\left(\frac{\partial \bar{\zeta }}{\partial y}+\beta \right)\frac{\partial \psi^{\prime }}{\partial x}. \end{array}$$

This together with the boundary condition

(34) $$\begin{array}{c} \left\{\begin{array}{cc} \psi^{\prime }=0\; & \mathrm{at}\;y=\pm 1,\\ \psi^{\prime }\;\mathrm{is}\;\mathrm{periodic}\;\mathrm{in}\;x, & \;\\ \frac{\partial \psi^{\prime }}{\partial z}=0\; & \mathrm{at}\;z=0,1, \end{array}\right. \end{array}$$

forms a linear system. Write (33) and (34) as

(35) $$\begin{array}{c} \frac{\partial \zeta^{\prime }}{\partial t}\equiv L_{\zeta }\psi^{\prime }, \end{array}$$

and write $\zeta^{\prime }$ together with (34) as

(36) $$\begin{array}{c} \zeta^{\prime }={\nabla_{H}}^{2}\psi^{\prime }+\frac{\partial \;}{\partial z}\left(S\frac{\partial \psi^{\prime }}{\partial z}\right)\equiv L\psi^{\prime }. \end{array}$$

Then, the linearized perturbation equation becomes

(37) $$\begin{array}{c} \frac{\partial \;}{\partial t}L\psi^{\prime }=L_{\zeta }\psi^{\prime }. \end{array}$$

As L is linear and independent from *t*, it commutes with ∂/∂t. We hence obtain the following linear dynamical system for the perturbation field $\psi^{\prime }$:

(38) $$\begin{array}{c} \frac{\partial \psi^{\prime }}{\partial t}=\left(L^{-1}\circ L_{\zeta }\right)\psi^{\prime }. \end{array}$$

The discretized version of (38) is denoted as:

(39) $$\begin{array}{c} \frac{du}{dt}=Au, \end{array}$$

where u is a vector of the values of $\psi^{\prime }$. Initialized with a vector $u_{0}$, its solution is

(40) $$\begin{array}{c} u=e^{At}u_{0}. \end{array}$$

**Figure 4:**
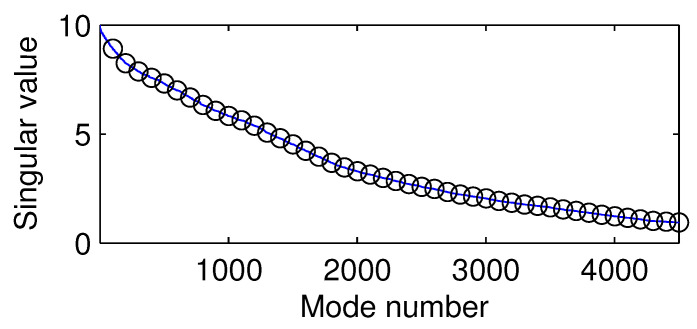
Singular value versus modal number.

The optimal perturbation corresponds to the largest singular value of the matrix $e^{At}$. To see this, it suffices to consider one particular time, say, t=1. Perform a singular value decomposition of $e^{A\cdot 1}$. Perturbations of the modal forms corresponding to singular values greater than 1 will grow. Here, the singular values become smaller than 1 after the modal number m>4333, as shown in Figure 4. In order to have the disturbance grow, we need to choose the modes with numbers lower than 4333.

**Figure 5:**
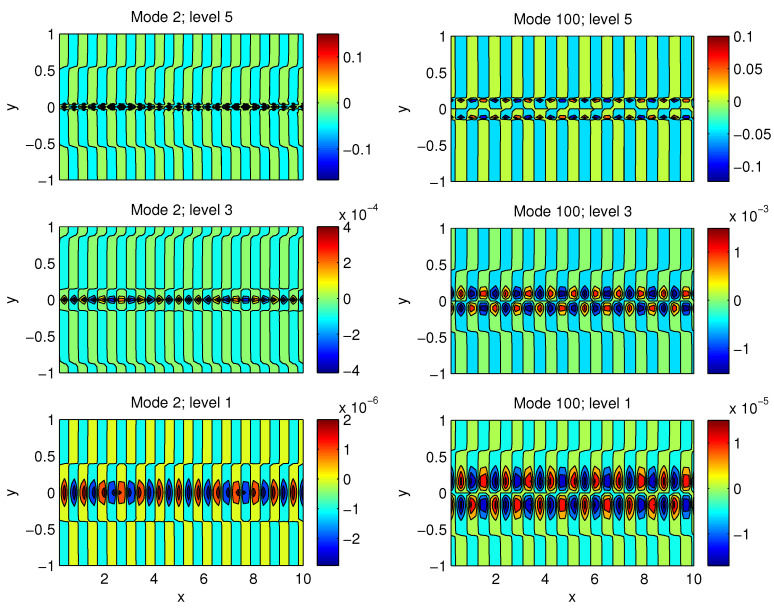
Perturbation modes.

**Figure 6:**
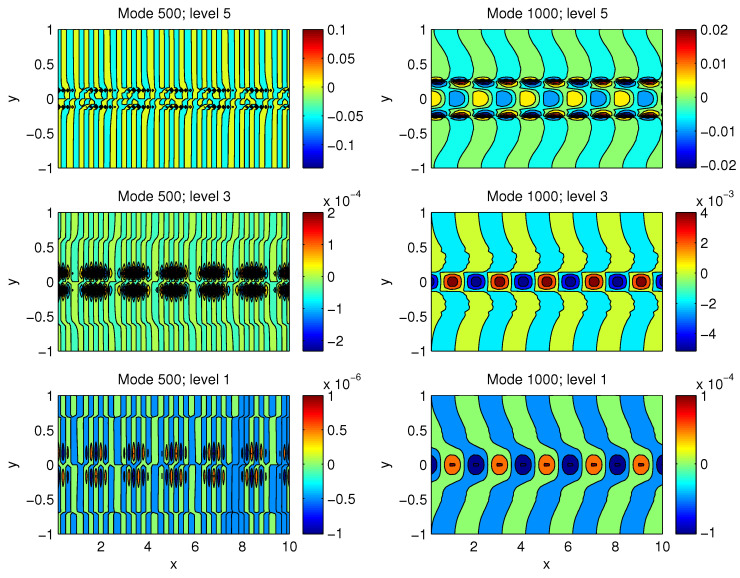
Perturbation modes (cont’d).

Displayed in Figure 5 and Figure 6 are modes 2, 100, 500, and 1000 (mode 1 is trivial). Clearly, they have different structures in both horizontal and vertical directions. Theoretically, the lower the modes, the more efficient the perturbation. However, since this is just a linear solution, the evolution after the initial perturbation may not grow as expected after nonlinearity takes effect. Here, we find that modes 100, 500, and 1000, among others, are satisfactory. In the following, we choose mode 1000 as the perturbation to initialize the system.

## 4. Reduced Model

### 4.1. Results of the Quasi-Geostrophic Model

**Figure 7:**
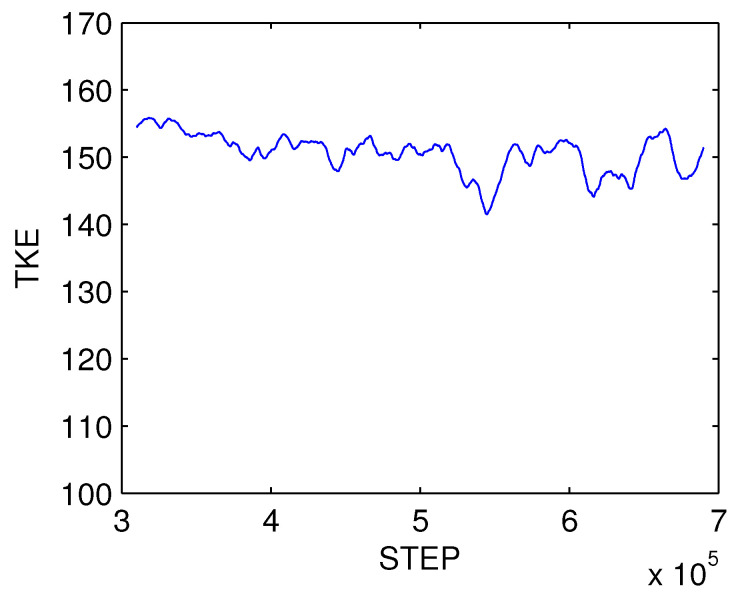
Evolution of total perturbation kinetic energy.

After initialization, the model reaches a quasi-equilibrium after some 400,000 steps. Figure 7 shows the evolution of total perturbation kinetic energy. Consider for our purpose the time interval between steps 500,000 and 650,000. We choose such an interval because (1) it is not too large; otherwise, too many processes may be involved and hence the model cannot be reduced much, (2) the processes during the interval appear to be stationary. Another reason is that the energetic cycle mimicks well that in the mid-latitude atmosphere. To see this, apply a multiscale window transform (MWT) to separate the process. MWT is a functional analysis tool developed by Liang and Anderson [24] which can decompose a function space into a direct sum of orthogonal subspaces, each with an exclusive range of scales, while preserving the local properties of the functions. Such a subspace is called a “scale window”. Originally, MWT is developed for a physically consistent expression of multiscale quadratic quantities, and hence to make multiscale energetics analysis possible. Traditionally, filters have been widely used for multiscale studies in atmospheric research, but, in a rigorous sense, the traditional filters are generally incapable of representing multiscale energy, which is a concept in phase space (it is connected to energy in the physical sense thanks to the Parseval identity), while the outputs of traditional filters are fields in physical space. Liang and Anderson [24] found that, for a class of specially designed orthogonal filters, there exists a transform-reconstruction pair, i.e., a pair of MWT and multiscale window reconstruction (MWR), just as Fourier transform and inverse Fourer transform. An MWR is just like a filtered quantity, while the corresponding MWT coefficient squared gives the energy on the scale window of concern. With MWT and MWR, it has been established that, for an atmospheric/oceanic flow, at each location, there exists a local Lorenz cycle consisting of three conservative processes. The resulting energy transfers have been referred to as canonical transfers; they all bear a Lie bracket form, in contrast to the classical emprically-obatained energy transfers. A comprehensive introduction of the theory is beyond the scope of this study; for details, see [25].

**Figure 8:**
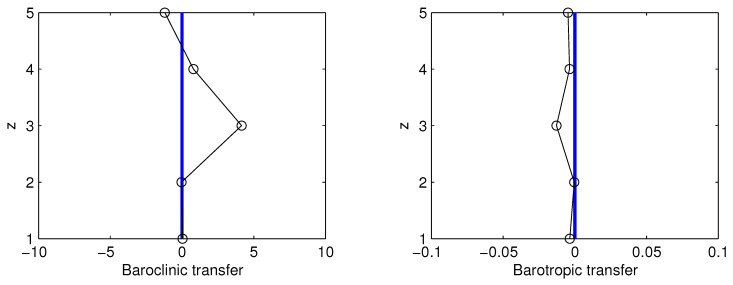
Barotropic and baroclinic canonical transfers from the mean state to the perturbation field.

Now, perform a two-scale window decomposition, and choose the longest scale to be the whole interval. (This can be done by setting the lowest scale window index to be zero; see [24]). With this setting, compute the canonical transfers using the localized multiscale energetics of Liang (2016a) [25]. The horizontally integrated canonical transfers are shown in Figure 8, where positive values indicate a transfer of energy from the mean to the perturbation. Clearly, here the transfer of available potential energy overwhelmingly dominates that of kinetic energy (two orders larger), and, on the whole, the former is cascaded downward (left panel), while the latter is transferred inversely upward (right panel). This seems to agree with what has been observed in the mid-latitude atmosphere (e.g., [14,15]).

### 4.2. Principal Component Analysis

**Figure 9:**
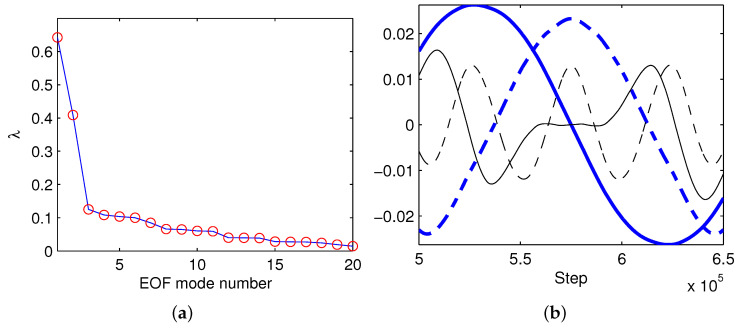
(**a**) eigenvalue λ vs. EOF mode number; (**b**) first four principal components: mode 1 (thick solid), mode 2 (thick dashed), mode 3 (thin solid), and mode 4 (thin dashed).

With the modeled 4D field ψ(x,y,z;t) on the chosen time interval, we perform a principal component (PC) analysis, or empirical orthogonal function (EOF) analysis as it is called. The eigenvalues λ are shown in Figure 9a. Obviously, the first three modes possess most of the variance. Displayed in Figure 9b are some of the corresponding PCs. It appears that the first and second PCs approximately are in quadrature phase; they should form a harmonic subsystem. The third and the fourth have similar frequencies, though that of the latter is a little higher.

### 4.3. Model Reduction

The EOF modes form an orthonormal basis for ψ. In this subsection, we use the basis to reduce the original governing Equation (15) into a low-dimensional dynamical system.

With the operator L as defined in (36), Equation (15) becomes

(41) $$\begin{array}{c} {\frac{\partial \psi }{\partial t}}^{\prime }=-L^{-1}\left[J\left(\psi^{\prime },L\psi^{\prime }\right)\right]-L^{-1}\left[U\frac{\partial L\psi^{\prime }}{\partial x}\right]-L^{-1}\left[\left(\beta +{\bar{\zeta }}_{y}\right){\frac{\partial \psi }{\partial x}}^{\prime }\right]. \end{array}$$

In the equation, $L^{-1}$ is the inverse of L. Expand ψ with ${\left\{e_{m}\right\}}_{m=1,2,\ldots }$ and truncate at m=4 to get:

(42) $$\begin{array}{c} \psi =\sum_{m=1}^{4}p_{m}\left(t\right)e_{m}\left(x,y,z\right). \end{array}$$

Substitution of this expansion into (41) yields

(43) $$\begin{array}{cc} & \sum_{m}\frac{dp_{m}}{dt}e_{m}=\\ & \;-L^{-1}\left[J\left(\sum_{i}p_{i}e_{i},L\sum_{j}p_{j}e_{j}\right)\right]-L^{-1}U\frac{\partial L\sum_{i}p_{i}e_{i}}{\partial x}-L^{-1}\left[\beta +{\bar{\zeta }}_{y}\right]\frac{\partial \sum_{i}p_{i}e_{i}}{\partial x}\\ & =-\sum_{i}\sum_{j}p_{i}p_{j}L^{-1}\left[J\left(e_{i},Le_{j}\right)\right]-\sum_{i}p_{i}L^{-1}\left(U\frac{\partial Le_{i}}{\partial x}\right)-\sum_{i}p_{i}L^{-1}\left[\left(\beta +{\bar{\zeta }}_{y}\right)\frac{\partial e_{i}}{\partial x}\right]. \end{array}$$

Since $\left\{e_{m}\right\}$ is orthonormal, taking an inner product on both sides with $e_{m}$ results in

(44) $$\begin{array}{ccc} \frac{dp_{m}}{dt} & = & -\sum_{i}\sum_{j}p_{i}p_{j}\langle e_{m},\;L^{-1}\left[J\left(e_{i},Le_{j}\right)\right]\rangle \\ & & -\sum_{i}p_{i}\langle e_{m},\;L^{-1}\left(U\frac{\partial Le_{j}}{\partial x}\right)\rangle \\ & & -\sum_{i}p_{i}\langle e_{m},\;L^{-1}\left[\left(\beta +{\bar{\zeta }}_{y}\right)\frac{\partial e_{i}}{\partial x}\right]\rangle \\ & = & \sum_{i}\sum_{j}\alpha_{m,i,j}^{N}p_{i}p_{j}+\sum_{i}\alpha_{m,i}^{L}p_{i},\;m,i,j=1,\ldots ,4, \end{array}$$

where

(45) $$\begin{array}{c} \alpha_{m,i,j}^{N}=-\langle e_{m},\;L^{-1}[J\left(e_{i},Le_{j}\right]\rangle \end{array}$$

are the quadratic term (nonlinear) coefficients, and

(46) $$\begin{array}{c} \alpha_{m,i}^{L}=-\langle e_{m},\;L^{-1}\left[U\frac{\partial Le_{i}}{\partial x}+\left(\beta +{\bar{\zeta }}_{y}\right)\frac{\partial e_{i}}{\partial x}\right]\rangle \end{array}$$

are the linear term coefficients.

By computation, the linear coefficients $\alpha_{m,i}^{L}$ are
$\alpha_{1,i}^{L}$−$3.4755100\times 10^{-2}$1.423829−$1.3951972\times 10^{-2}$0.1284307$\alpha_{2,i}^{L}$−1.417942−$1.3652847\times 10^{-2}$−0.1443377−$3.5396349\times 10^{-3}$$\alpha_{3,i}^{L}$−$1.3292501\times 10^{-2}$0.1588890$2.1233991\times 10^{-2}$0.4809141$\alpha_{4,i}^{L}$−0.1304244−$6.8064928\times 10^{-3}$−0.4592572$7.3776743\times 10^{-3}$
Likewise, from Equation (45), the coefficients for the quadratic terms are computed as follows:
$\alpha_{1,i,j}^{N}$ (m=1)


−$7.5820560\times 10^{-4}$−$3.2869954\times 10^{-2}$$3.5282248\times 10^{-3}$$4.9599465\times 10^{-3}$$4.1258920\times 10^{-2}$$1.6017430\times 10^{-3}$$4.2935433\times 10^{-3}$$4.6059955\times 10^{-5}$−$2.4720350\times 10^{-2}$$1.6584761\times 10^{-2}$−$9.0469969\times 10^{-3}$$7.8747235\times 10^{-3}$−$2.3384064\times 10^{-2}$−$9.9051173\times 10^{-4}$−$1.1057421\times 10^{-2}$−$5.0229015\times 10^{-4}$$\alpha_{2,i,j}^{N}$ (m=2)


−$2.2212272\times 10^{-3}$−$1.6904000\times 10^{-2}$−$2.1221174\times 10^{-3}$−$1.2588169\times 10^{-4}$$2.0407232\times 10^{-2}$−$1.0814944\times 10^{-3}$$3.0389655\times 10^{-3}$$1.2576354\times 10^{-3}$−$3.3598884\times 10^{-3}$−$2.4367530\times 10^{-2}$$2.1593377\times 10^{-3}$$4.7868756\times 10^{-3}$−$1.0774944\times 10^{-2}$−$4.2805336\times 10^{-3}$−$4.0617036\times 10^{-3}$−$2.7537413\times 10^{-4}$$\alpha_{3,i,j}^{N}$ (m=3)


−$9.9601485\times 10^{-3}$−$7.9435982\times 10^{-2}$−$1.4256314\times 10^{-2}$−$6.8288655\times 10^{-3}$$8.4469259\times 10^{-2}$−$1.6651559\times 10^{-3}$$6.7729242\times 10^{-2}$−$2.0513053\times 10^{-3}$$1.2046070\times 10^{-2}$−$6.1348621\times 10^{-2}$−$7.0710764\times 10^{-3}$$9.7337542\times 10^{-3}$−$1.4477096\times 10^{-3}$$1.6220149\times 10^{-2}$−$4.4799279\times 10^{-3}$$3.3035765\times 10^{-3}$$\alpha_{4,i,j}^{N}$ (m=4)


−$3.0739678\times 10^{-3}$−0.1182990$1.2369854\times 10^{-2}$$1.4575672\times 10^{-2}$0.1257915−$2.2905001\times 10^{-3}$−$2.0050334\times 10^{-2}$$9.9308938\times 10^{-3}$−$4.3914612\times 10^{-2}$$5.8852371\times 10^{-2}$$9.3064429\times 10^{-3}$−$3.0944983\times 10^{-2}$−$4.8473705\times 10^{-2}$−$4.0869847\times 10^{-2}$$7.3413953\times 10^{-2}$−$2.7747510\times 10^{-3}$

Note that in reconstructing ψ there is actually a mean part $\bar{\psi }\equiv p_{0}$ to be added. That is to say,

$$\psi =p_{0}+\sum_{i}p_{i}e_{i}.$$

Theoretically, this part should vanish in the system, but, in reality, it may not. If it is added to Equation (43), then

(47) $$\begin{array}{c} \sum_{m}\frac{dp_{m}}{dt}e_{m}=\\ \;-L^{-1}\left[J\left(\sum_{i}p_{i}e_{i},L\sum_{j}p_{j}e_{j}\right)\right]-L^{-1}U\frac{\partial L\sum_{i}p_{i}e_{i}}{\partial x}-L^{-1}\left[\beta +{\bar{\zeta }}_{y}\right]\frac{\partial \sum_{i}p_{i}e_{i}}{\partial x}\\ \;-L^{-1}J\left(p_{0},L\sum_{j}p_{j}e_{j}\right)-L^{-1}J\left(\sum_{i}p_{i}e_{i},Lp_{0}\right)-L^{-1}U\frac{\partial Lp_{0}}{\partial x}-L^{-1}\left[\left(\beta +{\bar{\zeta }}_{y}\right)\frac{\partial p_{0}}{\partial x}\right]. \end{array}$$

The second line is the new term in comparison to the original one. Thus, the following

$$-\langle e_{m},\;L^{-1}\left[J\left(p_{0},Le_{i}\right)+J\left(e_{i},Lp_{0}\right)\right]\rangle$$

should be added to the above coefficients $\alpha^{L}$. In addition, there exists an nonautonomous term

$$b_{m}=-\langle e_{m},\;L^{-1}\left[U\frac{\partial Lp_{0}}{\partial x}+\left(\beta +{\bar{\zeta }}_{y}\right)\frac{\partial p_{0}}{\partial x}\right]\rangle .$$

However, here it is shown that all these are negligible. Thus, it is adequate to use the above autonomous system in the following studies.

## 5. Information Flow between the Scales of the Model Atmosphere

**Figure 10:**
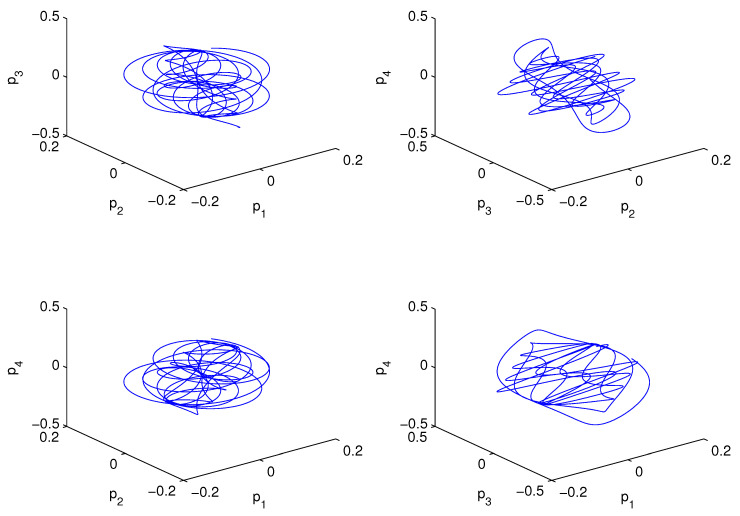
The trajectory of a sample path.

As we showed above, the interactions among the first four EOF modes can be utilized to study the multiscale interactions typical in the problem of concern, as the modes occur on different time scales. In order to examine the information flow between the modes, we make random draws for $\left(p_{1},p_{2},p_{3},p_{4}\right)$ from a pool of values, and then, starting from these initial conditions, run forward the system to generate an ensemble of solutions. Assume that the initial values obey a normal distribution with a mean vector (0.1,0.1,0.1,0.1) and a 4×4 identity covariance matrix. Here, the variance is set rather small in order for the trajectories to stay under effective control. The sample space is assumed to be [−6,6]×[−6,6]×[−6,6]×[−6,6], which makes sense if we do not make too long an integration, as made evident in Figure 10, where the trajectory of a sample path is plotted. The space is discretized using a spacing Δ=0.2 (the same for the four dimensions), and the probability density functions are then estimated at each time step by counting the bins in the coarse-grained space.

To compute the information flows among the four components, recall the deterministic version of Equation (2)

(48) $$\begin{array}{c} T_{j\to i}=-\int_{R^{n}}\rho_{j|i}\left(p_{j}|p_{i}\right)\frac{\partial F_{i}\rho_{\setminus \;j}}{\partial p_{i}}dp. \end{array}$$

**Figure 11:**
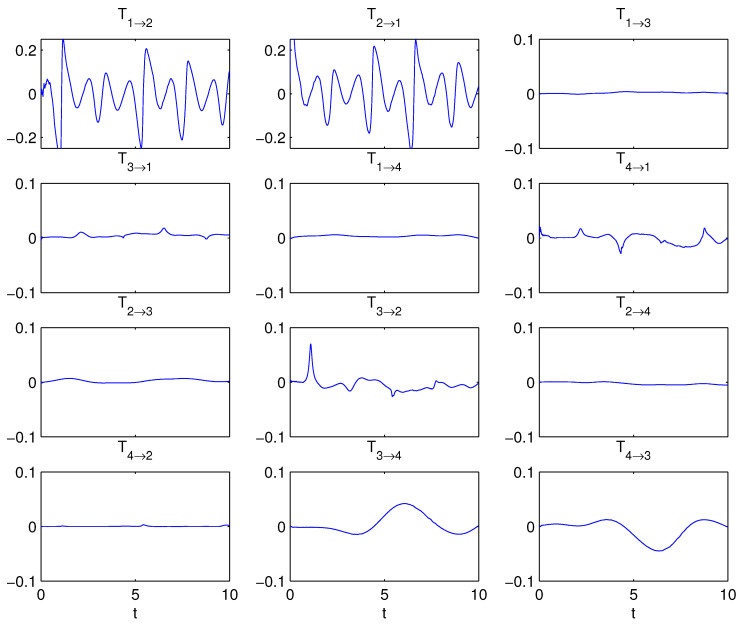
The computed information flows among the components of the reduced system. Note the range scale for the first two subplots ($T_{1\to 2}$ and $T_{2\to 1}$) is twice that for the others.

When the system is initialized with values in a rather limited domain, the integration can be easily evaluated. In this study, $R^{4}$ is replaced by [−6,6]×[−6,6]×[−6,6]×[−6,6]. The computed information flow evolutions vs. time are plotted in Figure 11.

For a system with four components, by expectation, there are in general 4×3=12 information flows. As we have shown before, the four components make two pairs, i.e., $\left(p_{1},p_{2}\right)$ and $\left(p_{3},p_{4}\right)$, which essentially represent two scales. Thus, the cross-scale information flows are those between modes (1,2) and modes (3,4). In Figure 11, $T_{1\to 2}$ and $T_{2\to 1}$ are overwhelmingly large (note the different scale range in the first two subplots); second to them are $T_{3\to 4}$, $T_{4\to 3}$. By the property of causality (ideally nonzero information flow implies causality), that is to say, $p_{1}$ and $p_{2}$ are mutually causal, and so are $p_{3}$ and $p_{4}$. These are the information flows within their respective scales. These causal patterns are similar to that between the displacement and linear momentum of a harmonic oscillator, as is shown in Liang [36]. From the table of $\alpha_{m,i}^{L}$ indeed to the first order, the system is like

(49) $$\begin{array}{c} \frac{d\;}{dt}\left(\begin{array}{c} p_{1}\\ p_{2} \end{array}\right)=\left(\begin{array}{cc} 0 & \;1.4\\ -1.4 & 0 \end{array}\right)\left(\begin{array}{c} p_{1}\\ p_{2} \end{array}\right) \end{array}$$

just as the computed $T_{1\to 2}$ and $T_{2\to 1}$ would imply.

The other information flows are interscale. Strictly speaking, there exist flows in both directions (small-scale⟶large-scale and large-scale⟶small-scale). However, by observation, $|T_{4\to 1}|$ and $|T_{3\to 2}|$ are much larger than others. This asymmetric flow structure indicates that the causation between the scales are dominantly one-way, i.e., from higher frequency modes (modes 3 and 4) to lower frequency modes, modes 1 and 2.

It should be mentioned that what has been solved is actually the QG equation for the perturbation field; the mean flow is not included in the four components $\left(p_{1},p_{2},p_{3},p_{4}\right)$ of the reduced system. However, the influence has been embedded in the system. Here, we give it an evaluation.

For notational convenience, let $p_{0}$ denote the “mean component.” Since here the mean flow is prescribed, it does not vary in time, so there is no way to examine the influence of other components on it. That is to say, there is no base to study $T_{i\to 0}$, but nonetheless we can evaluate $T_{0\to i}$, i=1,2,3,4. We know, from the Bayes’ rule, that

(50) $$\begin{array}{c} \rho_{0|i}\left(p_{0}|p_{i}\right)=\frac{\rho \left(p_{i}|p_{0}\right)\rho_{0}\left(p_{0}\right)}{\rho_{i}\left(p_{i}\right)}. \end{array}$$

Since the mean flow is prescribed, it is certain; $\rho (p_{i}|p_{0})$ is hence in fact $\rho_{i}\left(p_{i}\right)$. Thus, the whole term is equal to 1. This substituted into (48) yields

(51) $$\begin{array}{ccc} T_{0\to i} & = & -\int_{R^{n}}\rho_{0|i}\frac{\partial F_{i}\rho_{\setminus \;0}}{\partial p_{i}}dp\\ & = & -\int_{R^{n}}\frac{\partial F_{i}\rho }{\partial p_{i}}dp\\ & = & 0 \end{array}$$

by the compactness of ρ. That is to say, the information flow between the mean flow and the higher frequency components, if it exists, cannot be toward higher modes. In other words, if existing, it must be one way, i.e., in the direction upward toward the mean.

It should be emphasized that, generally, the mean flow should also have a distribution, and hence the information flow may not be this easy to evaluate. However, in this case, as we have shown in the preceding section, the variation around the mean is so small that it can be neglected in forming the low-dimensional system. Anyhow, for this particular case, by computation the information flow, and hence causation, is essentially one-way, i.e., from high frequency modes to low frequency modes.

We want to mention that here EOF analysis has been used to reduce the model order. The advantages of using it include its orthonormality, the maximization of variance toward lowest modes, etc. The limitations of this approach are also well known. The most serious one is that the EOF modes may not be real modes in the physical sense. Here, what we are investigating is the information exchange between processes on different temporal scales, and, fortunately, the principal components of the lowest modes do reflect such temporal variabilities (Figure 9). However, in a more general situation, this may not be true. We hope some advanced methods, such as the recently developed method by Majda and Qi [50] to efficiently reduce models, can help here.

An alternative approach is to use Equation (4) to estimate from data, rather than directly compute, the information flow, and hence avoid solving a large-dimensional Liouville equation (the curse of dimensionality). However, here comes another issue: Theorem 3 relies on the assumption of Gaussianity. Though (4) has also been successfully applied to some highly nonlinear systems, e.g., the chaotic anticipatory system in [41] (see [31]), caution should be used, as non-Gaussianity may appear significant in realistic atmospheres. However, anyhow, these are topics for future studies; here as the first step, we only consider what we have generated with the QG model.

## 6. Discussion and Conclusions

How processes on different scales interact to form weather and climate patterns is one of the central issues in dynamic meteorology. Traditionally, it is studied by diagnosing the exchange of energy (such as the Lorenz cycle), or, equivalently, momentum/angular momentum, between the scales. However, it has long been realized that just multiscale energetics based on the governing equation may not be enough. In a nonlinear dynamical system, as time moves on, two highly correlated events may soon lose correlation, while, on the other hand, two completely irrelevant events could turn out to be correlated in the end. As remarked by Corning [51], the underlying causal efficacy may actually be missing in the equations or “rules”. In addition, in the classical multiscale formalism, cyclogenesis is driven by Reynolds stress, which is essentially the linear correlation between the perturbation fields. As we proved earlier on, while causation implies correlation, correlation does not necessarily imply causation. That said, the traditional perspective on the problem may be limited.

In physics, entropy is another concept as important as energy. The transference of entropy results in a flow of information, but how information flows or transfers across scales has been overlooked in dynamic meteorology, in contrast to the extensively studied energy transfer. Recently, information flow has been rigorously formulated in the framework of dynamical systems; it proves to satisfy the “principle of nil causality” (see [36]), an observational fact which people endeavor to verify in real applications. In this study, this formalism is applied to study the information flow among the scales within a three-dimensional quasigeostrophic (QG) circulation. The basic flow is a zonal jet mimicking the atmospheric jet stream. We chose a period when the system is in equilibrium with an energetic scenario typical of a mid-latitude atmosphere: the mean state is releasing available potential energy to eddies, while the latter feeds kinetic energy back to the mean state. We first solved the 3D QG equation; then, for the period of concern, performed a principal component analysis and obtained the EOF modes to construct a basis. It has been shown that these modes characterize the desired temporal scales. The state variable, i.e., streamfunction, is then expanded with the aid of the basis, and the expansion is truncated at the fourth term. By inverting a 3D elliptic differential operator, the QG equation is converted into a four-dimensional dynamical system. The study of the information flows among the scales is then converted into the investigation of the information flows among the components of the low-dimensional system.

Initialized with an ensemble of streamfunctions drawn randomly according to a normal distribution, the system is integrated forward and, at each step, a probability density function is estimated, which, by Formula (2), allows us to obtain the desired information flow pairs. By computation mode 1 and mode 2, which represent the long temporal scale, are mutually causal, functioning like the components of a 2D harmonic oscillator; this is also the case for mode 3 and mode 4 that represent the motion on a short scale. These are the information flows within their respective scales. The interscale flows are significant only for that from mode 4 to mode 1 and that from mode 3 to mode 2, i.e., from modal pair (3,4) to modal pair (1,2). In addition, the possibility that the mean state has information flow to these four modes are excluded. That is to say, for this particular problem, the information flow is mostly one-way—from higher frequency modes to lower frequency modes. Hence, for this particular problem, underlying the multiscale interaction is mostly a bottom-up causation.

The bottom-up causation, or the information flow from the low levels to higher levels, is actually seen in many natural and social phenomena. In investigating the transition in biological complexity, for example, a reductionist will view the emergence of new, higher level, aggregate entities as a result of lower level entities (e.g., [52,53,54]). Similarly, it is found that some simple computer networks may transit from a low traffic state to a high congestion state, entailing a flow of information from a combination of independent objects to a collective pattern representing a higher level of organization. Most of all, in statistical physics [55,56], bottom-up causation lays for it the theoretical foundation, based on which the macroscopic thermodynamic properties can be tracked back to random molecular motions.

However, we did not exclude the existence of information flow the other way around; it is just weak by comparison in this example. Top-down causation has been found in many fields. For example, in community ecology, it has been argued that host community-level structures may determine the disease dynamics and hence control the constituent populations (e.g., [57]). Nonetheless, here we showed that a prescribed mean flow seems to be unlikely to have information flow to the anomalies.

Of course, the result here is just for a particular case with a reduced-order model; in reality, the problem could be very complicated, depending on the stage where the evolving state is. In addition, for simplicity, we have adopted a rigid-lid assumption on the top, and an idealized boundary condition ($\frac{\partial \psi }{\partial z}=0$, i.e., no density perturbation) at the bottom, although the simplified model does reproduce the desired downward transfer of available potential energy and upward kinetic energy. Nonetheless, the resulting interaction scenario is encouraging, in agreement with those in complex systems, although it is quite different from the corresponding energetic cycle. This result, though preliminary at this stage, may help better understand the mean flow–eddy interaction, gain deeper insight into the phenomena such as cyclogenesis, atmospheric blocking, sudden stratospheric warming, to name a few. On the other hand, the asymmetric causation (mostly bottom-up) provides an observational basis for the parameterization of the subgrid processes in numerical models, such as the stochastic closure scheme of Majda et al. [58]. All of these are interesting and deserve further investigation. We want to emphasize that information flow is a large field in atmospheric research, and this present study makes only a first attempt; much is yet to be explored in the future.

## Figures and Tables

**Table 1:** Model parameters.

Mesh	50×51×5
Δx	0.2
Δy	0.04
Δz	0.2
Δt	0.02
β	1
$Z(z_{k})$	0.2, 0.2, 0.6, 1, 1
$S(z_{k})$	0.943, 0.886, 0.828, 0.771, 0.714
